# Mortality and cancer incidence following occupational radiation exposure: third analysis of the National Registry for Radiation Workers

**DOI:** 10.1038/sj.bjc.6604825

**Published:** 2009-01-06

**Authors:** C R Muirhead, J A O'Hagan, R G E Haylock, M A Phillipson, T Willcock, G L C Berridge, W Zhang

**Affiliations:** 1 Health Protection Agency, Centre for Radiation, Chemical and Environmental Hazards, Chilton, Didcot, Oxon OX11 0RQ, UK

**Keywords:** cancer incidence, epidemiology, mortality, radiation, workers

## Abstract

Mortality and cancer incidence were studied in the National Registry for Radiation Workers in, relative to earlier analyses, an enlarged cohort of 174 541 persons, with longer follow-up (to 2001) and, for the first time, cancer registration data. SMRs for all causes and all malignant neoplasms were 81 and 84 respectively, demonstrating a ‘healthy worker effect’. Within the cohort, mortality and incidence from both leukaemia excluding CLL and the grouping of all malignant neoplasms excluding leukaemia increased to a statistically significant extent with increasing radiation dose. Estimates of the trend in risk with dose were similar to those for the Japanese A-bomb survivors, with 90% confidence intervals that excluded both risks more than 2–3 times greater than the A-bomb values and no raised risk. Some evidence of an increasing trend with dose in mortality from all circulatory diseases may, at least partly, be due to confounding by smoking. This analysis provides the most precise estimates to date of mortality and cancer risks following occupational radiation exposure and strengthens the evidence for raised risks from these exposures. The cancer risk estimates are consistent with values used to set radiation protection standards.

Estimates of the long-term health risks from ionising radiation are based largely on studies of the survivors of the atomic bombings in Japan and of groups exposed for medical reasons (NRC, 2006; ICRP, 2007; UNSCEAR, 2008). In view of the desirability of obtaining data relevant to protracted or low-dose radiation exposures, the National Radiological Protection Board (now the Radiation Protection Division of the Health Protection Agency) started the National Registry for Radiation Workers (NRRW) in 1976. Two earlier NRRW analyses (Kendall *et al*, 1992; Muirhead *et al*, 1999a, 1999b) found a strong ‘Healthy Worker Effect’ (HWE). When cancer mortality was analysed in relation to external radiation dose, the data were consistent both with existing radiation risk estimates and – for the most part – with the absence of an association. However, after excluding CLL which may not be radiation-inducible (UNSCEAR, 2008), there was borderline evidence in the second analysis (NRRW-2) of an increasing trend with dose in leukaemia mortality.

This paper summarises a third analysis (NRRW-3) that provides more precise information on the risks of occupational radiation exposure based on cancer registrations as well as mortality, data from an enlarged cohort of 174 541 workers, and a further 9 years of follow-up relative to NRRW-2. Further details are given by Muirhead *et al* (2009).

## Materials and methods

### Definition of cohort

The study population contains persons occupationally exposed to ionising radiation and for whom radiation dose records were kept. Data collected from employers consist of individual identifiers, factors such as date of birth, gender and industrial classification, and radiation dose histories. These data were audited prior to their inclusion in the analysis. Table 1 shows the study population by first employer and lifetime dose. All of these employers were included in NRRW-2. For some employers, the cohort is restricted to persons who undertook radiation work on or after 1 January 1976, when the NRRW was set up. However, earlier radiation workers are included in most instances. The NRRW-3 cohort contains about 50 000 more workers than before, namely:

for most employers, persons who started radiation work during 1991–1999;
workers who ceased employment at BNFL Capenhurst and Springfields before 1976;
Ministry of Defence radiation workers who ceased employment before 1977;
workers at British Energy Generation/Magnox Electric's Dungeness A and B power stations during 1965–1990.

Radiation workers are given the opportunity to refuse to participate in the NRRW. The refusal rate varied across organisations, but overall it was only about 1%.

This analysis – as before – focuses on doses from penetrating radiation at the surface of the body, estimated using personal dosemeters. Most of the doses are associated with X-rays and *γ*-rays, together – to a lesser extent – with *β* particles and neutrons. As doses were recorded primarily to ensure compliance with dose limits or constraints, corrections were applied to arrive at more accurate dose estimates (Muirhead *et al*, 1999b). The collective external dose was 4348 person Sv (Table 1), compared with 3810 person Sv from NRRW-2. The mean lifetime dose was 24.9 mSv overall, but varied considerably between employers. Six percent of workers had a lifetime dose of 100 mSv or more; they contributed 59% of the collective dose. Estimates of doses from internal emitters (i.e., radionuclides which have been inhaled or ingested) were not generally available and could not be used here, but workers monitored for potential exposure were identified.

The distribution of year of birth peaked between the late 1940s and the early 1960s (Muirhead *et al*, 2009). Just fewer than 10% of all workers were female; they tended to be born later than male workers and to have lower mean lifetime doses.

### Follow-up

Information on mortality, cancer registrations and emigrations was obtained from the NHS Central Registers (NHSCRs) for England and Wales and for Scotland, plus regional offices covering other parts of Britain and Ireland. Vital status checks were conducted at the Department for Work and Pensions and cross-checks were undertaken together with other research groups (Muirhead *et al*, 2009).

The end of follow-up was 31 December 2001. This date was chosen to ensure that complete personal and dose information was available up to at least 2 years previously, as 2 years was the shortest lag period used in the radiation analyses. At the end of follow-up, 28 320 out of the 174 541 cohort members were recorded as having died, 4579 were recorded as having emigrated and 1036 could not be traced satisfactorily.

### Statistical methods

The methods are similar to those used previously. The start of follow-up for each worker was the latest of: the date of start of radiation work with a participating employer (plus, in some instances, a lag of 2 or 10 years), the date from which full dose data were available, or 1 January 1955. The end of follow-up was the date of death or emigration (or cancer registration for the incidence analysis), the worker's 85th birthday, or 1 January 2002, whichever was earliest.

The analysis consisted of two parts:

#### External analysis:

Mortality was compared with rates for the general population of England and Wales by calculating standardised mortality ratios (SMRs), expressed as percentages. Thus an SMR of 100 denotes equality with national rates. Tests for trends and heterogeneity in SMRs were based on *χ*^2^ statistics (Breslow and Day, 1987). The external analysis was based on the underlying cause of death, coded according to the International Classification of Diseases (ICD)'s 9th revision (WHO, 1977). For disease groupings whose ICD codes varied between revisions, rates were bridge-coded. Mortality rates specific to social classes I and III were used in analyses of non-industrial and industrial workers, respectively. The external analysis was performed only for mortality, as cancer incidence, being based on a combination of registration and mortality data (see below), could not be compared with national cancer registration rates.

#### Internal analysis:

Because of a likely HWE, greater emphasis was placed on analyses internal to the cohort. Mortality and cancer incidence were studied in relation to dose after adjusting – through stratification – for age (in 5-year groups), gender, calendar period (1955-, 1960-, …, 1995, 2000–2001), industrial classification (industrial/non-industrial/unknown) and first employer (see Muirhead *et al*, 2009). Within each stratum, the number of deaths or cases expected in each category for cumulative external dose (0−, 10−, 20−, 50−, 100−, 200−, 400+ mSv) was calculated, conditional on the total over all dose categories and presuming no effect of dose. To allow for a latent period in any radiation effect and a particularly strong HWE soon after starting work, the first 2 years of follow-up following initial exposure were excluded when analysing leukaemia whereas the first 10 years were excluded for other cancers and deaths; doses were also lagged by 2 and 10 years respectively. Other lag periods were considered (Muirhead *et al*, 2009).

Following the lag period, the excess relative risk (ERR) – that is, the relative risk minus one – was modelled as a linear function of dose. The ERR per Sv was estimated by maximum likelihood (Little *et al*, 1993) and confidence intervals (CIs) were calculated and tests for trend in risk with dose were conducted using a score statistic (Darby and Reissland, 1981; Little *et al*, 1993). Particular weight was given to one-sided tests for any increase in risk with increasing dose, but two-sided tests were also conducted.

The cancer incidence analyses used the earliest cancer mentioned on a registration or a death certificate, except that:

leukaemia, multiple myeloma or lymphoma was selected ahead of other cancers, with the corresponding earliest date chosen;
non-melanoma skin cancer (NMSC) was selected only if no other malignancies were listed or if death was from a tumour of an unspecified site or a secondary cancer;
malignancies were selected in preference to benign conditions.

Points (i) and (iii) were also adopted for the mortality analyses.

## Results

### Mortality – external analysis

Data were available for 26 731 deaths during 3.9 million person-years of follow-up. Table 2 shows a strong HWE. Without adjustment for social class, the all-cause SMR was 81 (95% CI 80–82); the corresponding value for all malignant neoplasms was 84 (95% CI 82–86). The SMR for industrial workers was nearly 50% higher than that of non-industrial workers, but this difference was less marked using social class-specific rates. After adjusting for social class, there was still a strong HWE and, both for all causes and all malignant neoplasms, the social class-adjusted SMRs were very similar among men and women (Table 2). The SMRs for all causes and all malignant neoplasms decreased with increasing duration of radiation work, particularly for durations of at least 30 years, but there was less evidence for a decrease after adjusting for social class (Muirhead *et al*, 2009). All-cause SMRs by first employer generally varied between 63 and 90; the corresponding range using social class-adjusted rates was 72–90 (Muirhead *et al*, 2009).

For most specific causes of death, SMRs were less than 100 (Supplementary Table S1 on website). For common causes – particularly those related to smoking – this reduction was usually statistically significant. There were only a few causes of death with SMRs above 100. For thyroid cancer (unlagged and lagged SMRs of 110 and 123, respectively), testicular cancer (unlagged 103, lagged 65) and all uterine cancers combined (unlagged 85, lagged 102), the findings were consistent with national rates. In contrast, pleural cancer mortality was statistically significantly raised (unlagged SMR 209, lagged 207).

### Mortality – internal analysis

Supplementary Table S2 shows results from analyses that looked for trends in mortality with external radiation dose. Here, unlike Supplementary Table S1, the expected numbers were calculated internally to the cohort. There was borderline evidence of an increasing trend in total mortality with increasing dose from a one-sided test (*P*=0.049); the corresponding evidence from a two-sided test was weak (*P*=0.098). The evidence for this trend related mainly to cancer mortality; there was no statistically significant trend with dose in total mortality from other known causes, and the estimated ERR was lower than that for all causes combined. There was evidence of an increasing trend with dose in mortality from cancer overall from a one-sided test (*P*=0.036), although the evidence from a two-sided test was weaker (*P*=0.073); the ERR per Sv was 0.279 (90% CI 0.02, 0.56). Omitting leukaemia gave similar results.

Of the 28 non-overlapping groupings of cancers (and considering all liver cancer other than secondaries rather than primary liver cancer) in Supplementary Table S2, the estimated ERR per Sv was positive in 19 and negative in nine instances. Using a one-sided test at the 5% level, there were statistically significant increasing trends with dose in mortality from rectal cancer (*P*=0.027), laryngeal cancer (*P*=0.026), all uterine cancers (*P*=0.016) and leukaemia excluding CLL (*P*=0.042). In contrast, there was no evidence of a dose trend for all leukaemias combined (one-sided *P*=0.225). Only for all uterine cancers (*P*=0.03) was the estimated trend in mortality with dose significant at the 5% level using a two-sided test. There was no evidence for any cancer of a decreasing trend in mortality with increasing dose, using either a one-sided test for a decreasing trend or a two-sided test.

Among leukaemia subtypes, a one-sided test (*P*=0.027) indicated an increasing trend with dose in chronic myeloid leukaemia (CML) mortality; the two-sided *P*-value was 0.054 (Supplementary Table S3). Although not statistically significant, the estimated ERR per Sv was greater than zero for acute myeloid (AML) and acute lymphatic leukaemia (ALL) but less than zero for CLL, even when – as in Vrijheid *et al* (2008) – a 10-year lag was used (ERR per Sv <−1.929, 90% CI <−1.93, 1.83).

There was a statistically significant increasing trend with dose in mortality from all circulatory diseases combined from a one-sided test (*P*=0.03); a two-sided test gave *P*=0.059. Much of the evidence for this trend related to coronary heart disease (CHD) (one-sided *P*=0.053, two-sided *P*=0.105). When CHD was analysed together with other non-malignant diseases that are strongly related to smoking, there was no evidence of an increasing trend with dose. Indeed, there was very strong evidence (two-sided *P*=0.001) that mortality from bronchitis, emphysema and chronic obstructive disease decreased with increasing dose. For respiratory diseases unrelated to smoking, the one-sided *P*-value for an increasing trend with dose was 0.04, but 0.079 from a two-sided test.

### Cancer incidence – internal analysis

For malignancies overall, there were 11 165 cases (compared with 7684 deaths) and strong evidence of an increasing trend in total incidence with dose from both one-sided (*P*=0.018) and two-sided (*P*=0.036) tests; ERR per Sv 0.281 (90% CI 0.06, 0.53). Results were similar when either leukaemia or NMSC was omitted. Of the 29 non-overlapping groupings of cancers (and considering all liver cancer other than secondaries rather than primary liver cancer) in Supplementary Table S4, the estimated ERR per Sv was positive in 19 and negative in 10 instances. There were statististically significant increasing trends with dose for rectal cancer (one-sided *P*=0.02), all skin cancers (*P*=0.01), NMSC (*P*=0.02), multiple myeloma (*P*=0.008) and leukaemia excluding CLL (*P*=0.03); except for the last of these, the trends were also significant using a two-sided test. The trends for all uterine cancers (*P*=0.057), thyroid cancer (*P*=0.079) and non-Hodgkin lymphoma (*P*=0.081) approached statistical significance based on a one- (but not a two-) sided test. There was a statistically significant increasing trend with dose for endometrial cancer (one-sided *P*=0.01). No cancer showed a statistically significant decreasing trend in risk with increasing dose, using either a one-sided test for a decreasing trend or a two-sided test.

Among leukaemia subtypes, there was evidence from both one- (*P*=0.011) and two-sided (*P*=0.022) tests of an increasing trend with dose in CML incidence (Supplementary Table S3). The estimated ERR was greater than zero for AML and ALL and less than zero for CLL, although none of the trends was statistically significant. The estimate for CLL was still less than zero using a 10-year lag (ERR per Sv −0.337, 90% CI −1.72, 3.1). There was no evidence for an increasing trend with dose in the incidence of all leukaemias combined, whereas there was evidence of such a trend after omitting CLL.

### Subsidiary analyses

Generally, the main findings did not change greatly when the format of the analyses was altered (Muirhead *et al*, 2009). For mortality from leukaemia excluding CLL but not for incidence, the variation in the ERR per Sv by attained age was statistically significant, whereas the data for the grouping of all malignant neoplasms excluding leukaemia were consistent with a constant ERR by attained age.

## Discussion

### General patterns of mortality

As in previous NRRW analyses, overall mortality was lower than expected from national rates. Although the overall magnitude of the HWE has changed little between analyses, SMRs have varied over the follow-up, with indications of a decrease among workers with at least 30 years radiation work. In the 15-country nuclear worker study which included many of the workers in NRRW-2, adjustment was made for duration of radiation work or employment, so as to allow for any ‘healthy worker survivor effect’, and led to a sizeable increase in the estimated ERR per Sv for all cancers other than leukaemia (Cardis *et al*, 2007; Vrijheid *et al*, 2007). However, a similar stratification by whether or not the duration of radiation work was at least 10 years tended to reduce estimates of the ERR in NRRW-3; furthermore, stratifying by whether or not the duration was at least 30 years had little impact (Muirhead *et al*, 2009).

### Leukaemia

**Figure 1:**
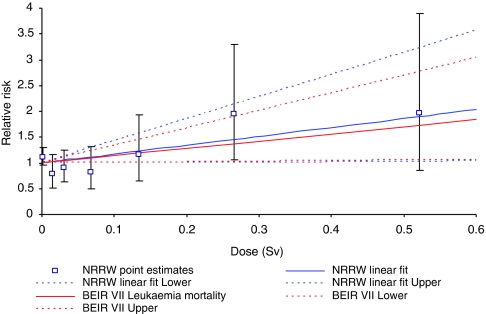
Trends with dose in relative risk (and 90% CI) for mortality from leukaemia excluding CLL.

Raised risks of leukaemia excluding CLL have been seen among the Japanese A-bomb survivors, radiotherapy patients and large groups of radiation workers (UNSCEAR, 2008), including previous NRRW analyses (Kendall *et al*, 1992; Muirhead *et al*, 1999a, 1999b). The number of deaths from leukaemia excluding CLL studied here is more than two times that in NRRW-2, whereas the corresponding number of cases here is nearly 2½ times the number of deaths in NRRW-2, hence the 90% CI for the ERR is about 40% narrower than before, Table 3 and Figure 1 show good agreement in the estimated ERR per Sv across NRRW-3 (for both mortality and incidence data), NRRW-2, the 15-country study and the Japanese A-bomb study. In particular, the findings from NRRW-3 are consistent with the dose reduction factor of two commonly used when extrapolating leukaemia risks among the Japanese A-bomb survivors down to low doses (ICRP, 2007). The 90% CI for the ERR (Table 3) indicates that the risk of leukaemia excluding CLL is greater than zero but is unlikely to be more than three times greater than that estimated by the BEIR VII Committee (NRC, 2006).

As before, the leukaemia subtype showing the strongest evidence of an association with radiation – from both mortality and incidence data – is CML. This has also been associated with radiation in the Japanese A-bomb survivors and some medically exposed groups (UNSCEAR, 2008). In contrast, there was no evidence of an association between CLL (either mortality or incidence) and radiation, even for a 10-year lag. This agrees with results from many other studies of radiation-exposed groups (UNSCEAR, 2008).

### All cancers other than leukaemia combined

Unlike previous NRRW analyses, NRRW-3 shows a statistically significant increasing trend with dose in both mortality and incidence for all malignant neoplasms other than leukaemia. The findings from the three NRRW analyses are mutually consistent, but have become progressively more precise. Relative to NRRW-2, the 90% CI for the ERR per Sv based on mortality (incidence) data is about 30% (40%) narrower. The NRRW-3 results are also consistent with those from the 15-country worker study (Table 3), although the estimated ERR is towards the lower end of the 90% CI from that study. The latter CI is considerably wider than that for NRRW-3, reflecting the higher ERR estimate in the 15-country study and its exclusion of some groups of workers with relatively high external doses because of potential internal exposure (Cardis *et al*, 2007). Stratifying the data according to whether a worker was ever internally monitored had little impact on our results. To reduce any possible confounding by smoking or asbestos exposure, Table 3 also shows results for all malignant neoplasms other than leukaemia, lung and pleural cancer; here the 90% CI from NRRW-3 lies within that of the 15-country study, so demonstrating consistency between the studies and the greater precision of NRRW-3.

**Figure 2:**
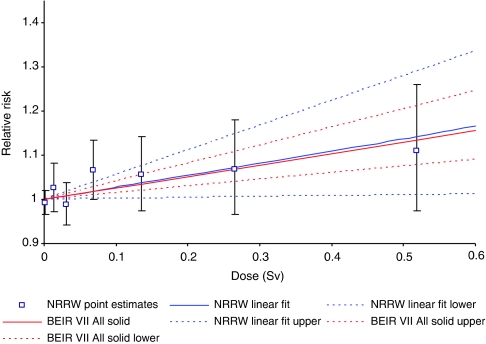
Trends with dose in relative risk (and 90% CI) for mortality from all malignant neoplasms excluding leukaemia.

**Figure 3:**
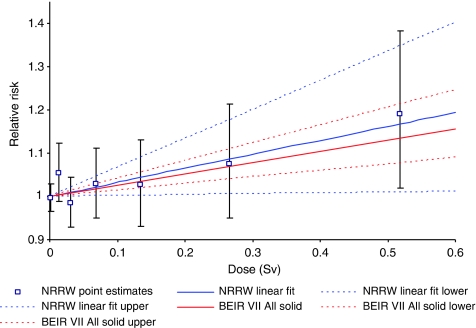
Trends with dose in relative risk (and 90% CI) for mortality from all malignant neoplasms excluding leukaemia, lung and pleural cancer.

Figure 2 shows good agreement between the ERR mortality estimates for all malignant neoplasms excluding leukaemia from NRRW-3 and the Japanese A-bomb study, based on a linear dose–response model. Similar inferences arise using incidence rather than mortality data (Table 3) and when excluding lung and pleural cancer (Figure 3). The 90% CI for all malignant neoplasms excluding leukaemia (Table 3) indicates that the risk from occupational radiation exposure is greater than zero but is unlikely to be more than about two times greater than that estimated from the A-bomb data using a linear dose–response model. An important theme in radiation protection is how to estimate cancer risks at low doses and low-dose rates using results from the A-bomb survivors, who received a wide range of doses acutely. ICRP (2007) recommended a dose and dose rate effectiveness factor (DDREF) of two when extrapolating from high doses and high-dose rates down to low doses and/or low-dose rates, while the BEIR VII Committee (NRC, 2006) derived a range for a low-dose extrapolation factor of (1.1, 2.3) with a central estimate of 1.5 (the BEIR VII risks cited in Table 3 and in Figures 2 and 3 for cancers other than leukaemia are based on a linear dose–response model and do not include a low-dose extrapolation factor). The NRRW-3 data are consistent with the BEIR VII factor and provide more evidence in favour of a solid cancer DDREF that is less than two rather than greater than two, but this latter possibility cannot be ruled out. The risk implied by NRRW-3 is unlikely to be more than four times greater than the A-bomb estimate incorporating a DDREF of two.

### Specific cancers

There was a statistically significantly increasing trend in multiple myeloma incidence (but not mortality) with dose. NRRW-3 contains nearly three times the number of myeloma deaths as NRRW-2 (which reported some evidence of a trend with dose in mortality), plus additional incident cases. However, the evidence for a dose trend here relates largely to small numbers among workers with relatively high doses (Supplementary Table S4). Other populations exposed to radiation have given mixed results for myeloma (UNSCEAR, 2008). Consequently, the interpretation of the NRRW-3 results is unclear.

Both NRRW-2 and NRRW-3 found a non-statistically significantly raised SMR for thyroid cancer, but no association between mortality and dose. As thyroid cancers are usually not fatal, the incidence data should be more informative. Although there was weak evidence of a trend with external dose in thyroid cancer incidence (one-sided *P*=0.079), this was driven primarily by two cases with a cumulative dose above 400 mSv. Relative to childhood exposures, adult exposures to either external radiation or radioiodine provide less evidence of a raised risk of thyroid cancer and suggest that any radiation risk would be smaller (UNSCEAR, 2008). The imprecise findings from NRRW-3 are consistent with this conclusion.

Except for skin cancer, the other cancers for which a dose trend was found here have rarely been associated with radiation in other studies (UNSCEAR, 2008). Furthermore, only the findings for rectal cancer and NMSC were based on large numbers of incident cases. Although NMSC has been linked to radiation in several populations (UNSCEAR, 2008), the relevant doses were mainly above those received here. Furthermore, the registration of NMSC is known to be poor compared to other cancers and some differential ascertainment cannot be ruled out; in addition, there is no information on ultraviolet radiation exposure, a key determinant of NMSC risk. Given that the estimated ERR for rectal cancer was imprecise and consistent with the corresponding estimate for all malignant neoplasms other than leukaemia (Supplementary Table S4), and that many types of cancer were studied, there is little evidence that rectal cancer is particularly radiosensitive.

Only for pleural cancer was there a statistically significant raised SMR, but there was no evidence of a dose trend in incidence or mortality. While there is no information in the NRRW on potential asbestos exposure, it is highly likely that this raised SMR is due to asbestos rather than radiation exposure.

### Non-cancer mortality

Studies of the Japanese A-bomb survivors and of patients who received high-dose radiotherapy to the heart have shown raised rates of heart disease (UNSCEAR, 2008). However, apart from the A-bomb study, other studies have not provided strong evidence of raised risks of circulatory diseases below doses of a few Sv (UNSCEAR, 2008). Occupational studies have yielded mixed results (e.g. Vrijheid *et al*, 2007; McGeoghegan *et al*, 2008). Furthermore, it was not possible in most worker analyses, including NRRW-3, to adjust for risk factors for circulatory diseases.

The estimated ERR per Sv for all circulatory diseases combined from NRRW-3 (0.251, 90% CI 0.03, 0.49) is comparable with the A-bomb estimate (Preston *et al*, 2003). Much of the evidence for a dose trend arises for CHD, which is particularly influenced by smoking. For each of CHD, aortic aneurysm, cerebrovascular disease, all circulatory diseases combined and lung cancer (but not for smoking-related respiratory diseases), the ratio of observed to expected numbers of deaths tended to increase with increasing dose, except for the highest dose group where this ratio falls below one (Supplementary Table S2). Also, the lack of evidence for a dose trend after adjusting for duration of radiation work (Muirhead *et al*, 2009) suggests that some feature of long-term radiation work other than radiation itself might influence circulatory disease risk, although the appropriate lag period is not known. In the absence of information on confounding factors, interpretation is difficult. The similar dose patterns in circulatory disease and lung cancer mortality suggest some confounding by smoking, but the direction and magnitude of this effect cannot be quantified.

## Figures and Tables

**Table 1:** Study population by lifetime dose and first employer^a^

	**Dose range (mSv)**						
**Employer**	**<10.0**	**10.0−**	**50.0−**	**100.0+**	**Total number of workers**	**Collective dose (person Sv)**	**Mean dose (mSv)**
Atomic Weapons Establishment	12 240	2157	281	162	14 840	122	8.2
British Energy Generation and Magnox Electric Ltd (England and Wales)	6313	5337	1132	613	13 395	323	24.1
British Energy Generation and Magnox Electric Ltd (Scotland)	1894	764	316	181	3155	71	22.7
British Nuclear Fuels plc (BNFL)	19 268	11 343	3972	5701	40 284	2160	53.6
GE Healthcare	2735	664	198	296	3893	122	31.4
HPA-RPD	207	51	15	8	281	4	14.2
MRC Harwell	348	14	2	0	364	1	2.1
Ministry of Defence	56 552	5878	1350	1129	64 909	522	8.0
Organisations using the HPA Personal Dosimetry Service	249	131	43	63	486	26	54.2
Rolls-Royce Submarines	2213	497	115	15	2840	26	9.0
Science and Technology Facilities Council	1709	600	83	36	2428	30	12.5
UK Atomic Energy Authority (UKAEA)	15 038	7966	2362	2300	27 666	940	34.0
Total	118 766	35 402	9869	10 504	174 541	4348	24.9

a Where possible, the most recent name for each participating organisation is used here. However, where the historical name provides a more concise means of identifying the relevant group of workers (eg., for BNFL and UKAEA), this name has been retained.

**Table 2:** Standardised mortality ratios (SMRs)^a^ by broad cause, gender and industrial classification

		**Number of deaths**		**Unadjusted**		**Social-class adjusted**	
**Gender**	**Industrial classification**	**Observed**	**Expected**	**SMR**	**95% CI^b^**	**SMR**	**95% CI**
*All causes*							
Both	All	26731	33014.00	81	80–82	84	83–85
	Industrial	18285	19660.37	93	92–94	82	80–83
	Non-industrial	8146	12950.43	63	62–64	90	88–92
Men	All	25841	31852.94	81	80–82	84	83–85
Women	All	890	1161.06	77	72–82	84	79–90
							
*χ*^*2*^ *for heterogeneity in SMR between men and women*				*2.77*		*0.01*	
							
*All malignant neoplasms*							
Both	All	8107	9666.63	84	82–86	82	81–84
	Industrial	5394	5640.72	96	93–98	80	78–82
	Non-industrial	2622	3905.75	67	65–70	88	85–91
Men	All	7752	9229.92	84	82–86	82	80–84
Women	All	355	436.71	81	73–90	84	76–93
*χ*^*2*^ *for heterogeneity in SMR between men and women*				*0.36*		*0.17*	

a Based on the general population of England and Wales.

b Confidence interval.

**Table 3:** Comparison of estimates of ERR per Sv (and 90% CI) for cancer in the NRRW, the 15-country nuclear worker study and the Japanese A-bomb survivors

	**Leukaemia excluding CLL**	**All malignant neoplasms excluding leukaemia**	**All malignant neoplasms excluding leukaemia, lung and pleura cancer**
*3rd NRRW analysis*			
Mortality	1.712 (0.06, 4.29)	0.275 (0.02, 0.56)	0.323 (0.02, 0.67)
Incidence	1.782 (0.17, 4.36)	0.266 (0.04, 0.51)	0.305 (0.05, 0.58)
			
*2nd NRRW analysis* (Muirhead *et al*, 1999a, 1999b)*: mortality*	2.55 (−0.03, 7.16)	0.09 (−0.28, 0.52)	0.17 (−0.26, 0.70)^a^
			
*15-country nuclear worker study* (Cardis *et al*, 2007)*: mortality*	1.93 (<0, 7.14)	0.97 (0.27, 1.80)	0.59 (−0.16, 1.51)
			
*Japanese A-bomb survivors*			
BEIR VII (NRC, 2006): mortality	1.4 (0.1, 3.4)^b^	0.26 (0.15, 0.41)^c^	—
BEIR VII (NRC, 2006): incidence	—	0.43^d^	—

a Based on data for all malignant neoplasms excluding leukaemia and lung cancer.

b Based on the low-dose component of a linear-quadratic dose–response model fitted to A-bomb data on mortality during 1950–2000. This estimate – as given by Cardis *et al* (2007) – applies to men exposed at ages of 30 years or more, at 15 years following exposure.

c Based on fitting a linear dose–response model to A-bomb data on solid cancer mortality during 1950–2000. This estimate – as given by Cardis *et al* (2007) – applies to men exposed at ages of 30 years or more, at an attained age of 50 years.

d Based on fitting a linear dose–response model to A-bomb data on the incidence of all solid cancers other than thyroid and non-melanoma skin cancers during 1958–98. The ERR estimate cited applies to men exposed at ages of 30 years or more, at an attained age of 50 years.
