# Supplementary material for: Mortality and cancer incidence following occupational radiation exposure: third analysis of the National Registry for Radiation Workers
Source: Br J Cancer. 2009 Jan 6;100(1):206–12. doi: 10.1038/sj.bjc.6604825 (PMC2634664; doi:10.1038/sj.bjc.6604825)
Supplement: Supplementary Table S1–S4 [file 6604825x1.doc]

Supplementary Tables (for the British Journal of Cancer’s website)

**TABLE S1 Standardised mortality ratios (SMRs) for different causes of death**

|  | ICD 9^th^ revision codes | Unlagged analysis | | | | | Lagged analysis ^1^ | | | | |
| --- | --- | --- | --- | --- | --- | --- | --- | --- | --- | --- | --- |
|  |  | Number of deaths | | |  | | Number of deaths | | |  | |
| Disease |  | Obs | Exp ^2^ | SMR | | 95% CI ^3^ | Obs | Exp | SMR | | 95% CI |
| All causes | 000-999 | 26731 | 33014 | 81 | | 80-82 | 23441 | 28404.41 | 83 | | 81-84 |
| All known causes excluding malignant neoplasms | 000-139 209-799.8 800-999.8 | 18097 | 23313.43 | 78 | | 76-79 | 15874 | 19889.15 | 80 | | 79-81 |
| All malignant neoplasms | 140-208 | 8107 | 9666.63 | 84 | | 82-86 | 7136 | 8486.91 | 84 | | 82-86 |
| All neoplasms | 140-239 | 8209 | 9779.55 | 84 | | 82-86 | 7227 | 8580.18 | 84 | | 82-86 |
| **Specific Malignancies** |  |  |  |  | |  |  |  |  | |  |
| Mouth, tongue and pharynx | 141, 143-148, 149.0 | 97 | 142.15 | 68 | | 55-83 | 85 | 124.01 | 69 | | 55-85 |
| Oesophagus | 150 | 349 | 414.38 | 84 | | 76-94 | 331 | 378.75 | 87 | | 78-97 |
| Stomach | 151 | 594 | 703.19 | 84 | | 78-92 | 498 | 598.91 | 83 | | 76-91 |
| Large intestine | 153, 159.0 | 616 | 690.56 | 89 | | 82-97 | 548 | 619.67 | 88 | | 81-96 |
| Rectum | 154.0-154.2 154.4-154.9 | 321 | 397.62 | 81 | | 72-90 | 275 | 350.35 | 78 | | 69-88 |
| Liver ^4^ | 155 | 94 | 120.22 | 78 | | 63-96 | 83 | 108.87 | 76 | | 61-95 |
| Primary liver | 155.0 | 44 | 66.77 | 66 | | 48-88 | 39 | 58.23 | 67 | | 48-92 |
| Gall bladder | 156 | 27 | 41.09 | 66 | | 43-96 | 27 | 34.77 | 78 | | 51-113 |
| Pancreas | 157 | 355 | 410.94 | 86 | | 78-96 | 320 | 363.86 | 88 | | 79-98 |
| Larynx | 161 | 65 | 89.03 | 73 | | 56-93 | 60 | 78.68 | 76 | | 58-98 |
| Trachea, bronchus and lung | 162 | 2433 | 3192.97 | 76 | | 73-79 | 2130 | 2790.19 | 76 | | 73-80 |
| Pleura | 163 | 112 | 53.68 | 209 | | 172-251 | 102 | 49.39 | 207 | | 168-251 |
| Bone | 170 | 14 | 23.7 | 59 | | 32-99 | 7 | 15.21 | 46 | | 18-95 |
| Connective and soft tissue | 171 | 35 | 42.37 | 83 | | 58-115 | 30 | 34.26 | 88 | | 59-125 |
| All skin | 172-173 | 113 | 123.5 | 91 | | 75-110 | 90 | 103.02 | 87 | | 70-107 |
| Female breast | 174 | 76 | 106.02 | 72 | | 56-90 | 61 | 85.51 | 71 | | 55-92 |
| Uterus | 179-182 | 23 | 26.95 | 85 | | 54-128 | 21 | 20.61 | 102 | | 63-156 |
| Ovary | 183 | 23 | 33.66 | 68 | | 43,103 | 18 | 27.47 | 66 | | 39-104 |
| Prostate | 185 | 629 | 641.26 | 98 | | 91-106 | 605 | 613.79 | 99 | | 91-107 |
| Testis | 186 | 33 | 31.9 | 103 | | 71-145 | 10 | 15.28 | 65 | | 31-120 |
| Bladder | 188, 189.3-189.9 | 291 | 341.52 | 85 | | 76-96 | 261 | 310.41 | 84 | | 74-95 |
| Kidney | 189.0-189.2 | 195 | 215.13 | 91 | | 78-104 | 170 | 190.24 | 89 | | 76-104 |
| Brain | 191-192, 224-225, 239.6 | 316 | 343.72 | 92 | | 82-103 | 261 | 275.07 | 95 | | 84-107 |
| Thyroid | 193 | 18 | 16.35 | 110 | | 65-174 | 17 | 13.81 | 123 | | 72-197 |
| Ill defined and secondary cancers | 195-196 | 634 | 677.46 | 94 | | 86-101 | 588 | 626.72 | 94 | | 86-102 |
| All lymphatic and haematopoietic | 200-208, 238.6 | 597 | 703.47 | 85 | | 78-92 | 508 ^5^ | 584.51 | 87 | | 80-95 |
| Non-Hodgkin lymphoma | 200, 202.2-202.3, 202.5-202.9 | 237 | 258.34 | 92 | | 80-104 | 206 | 222.47 | 93 | | 80-106 |
| Hodgkin lymphoma | 201 | 38 | 58.65 | 65 | | 46-89 | 28 | 34.45 | 81 | | 54-117 |
| Multiple myeloma | 203.0, 203.2-203.9, 238.6 | 106 | 129.43 | 82 | | 67-99 | 97 | 117.54 | 83 | | 67-101 |
| All leukaemia | 202.4, 203.1, 204-208 | 216 | 256.96 | 84 | | 73-96 | 215 | 248.99 | 86 | | 75-99 |
| Leukaemia excluding chronic lymphatic | 202.4, 203.1, 204.0, 204.2-207.7, 207.9-208.9 | 177 | 205.06 | 86 | | 74-100 | 176 | 197.58 | 89 | | 76-103 |
| All malignant neoplasms excluding leukaemia | 140-202.3, 202.5-203, 203.2-203.9 | 7891 | 9409.5 | 84 | | 82-86 | 6959 | 8276.81 | 84 | | 82-86 |
| All malignant neoplasms excluding leukaemia, lung and pleura | 140-161.9, 164-202.3, 202.5-203, 203.2-203.9 | 5346 | 6162.98 | 87 | | 84-89 | 4727 | 5437.27 | 87 | | 84-89 |
| Malignant neoplasms strongly related to smoking | 141, 143.0-149.0, 150.0-150.9, 157.0-157.9, 161.0-163.9, 188.0-189.9 | 3897 | 4859.33 | 80 | | 78-83 | 3459 | 4285.41 | 81 | | 78-83 |
| **Non-malignant diseases** |  |  |  |  | |  |  |  |  | |  |
| Infectious and parasitic diseases | 000-139 | 114 | 238.24 | 48 | | 39-57 | 99 | 175.26 | 56 | | 46-69 |
| Benign and ill-defined neoplasms | 209-239 | 102 | 112.84 | 90 | | 74-110 | 91 | 93.22 | 98 | | 79-120 |
| Nervous system diseases | 320-389 | 376 | 526.22 | 71 | | 64-79 | 356 | 434.99 | 82 | | 74-91 |
| Coronary heart disease | 410-414 | 8405 | 9974.11 | 84 | | 82-86 | 7447 | 8765.96 | 85 | | 83-87 |
| Bronchitis, emphysema and chronic obstructive disease | 491-492, 496, 519 | 1089 | 1763.94 | 62 | | 58-66 | 1015 | 1577.3 | 64 | | 60-68 |
| Aortic aneurysm | 441 | 505 | 551.31 | 92 | | 84-100 | 478 | 517.26 | 92 | | 84-101 |
| Non-malignant diseases strongly related to smoking | 410-414, 441, 491-492, 496, 519 | 9999 | 12289.39 | 81 | | 80-83 | 8940 | 10860.6 | 82 | | 81-84 |
| Circulatory diseases not strongly related to smoking | 390-409,  415-440, 442-459 | 12265 | 14762.38 | 83 | | 82-85 | 10961 | 12968.53 | 85 | | 83-86 |
| Cerebrovascular diseases | 430-438 | 2077 | 2594.49 | 80 | | 77-84 | 1905 | 2162.78 | 88 | | 84-92 |
| All circulatory diseases | 390-459 | 12265 | 14762.38 | 83 | | 82-85 | 10961 | 12968.53 | 85 | | 83-86 |
| Respiratory diseases (not smoking related) | 460-490, 493-495, 497-518 | 1028 | 1718.41 | 60 | | 56-64 | 960 | 1522.37 | 63 | | 59-67 |
| Digestive disease | 520-579 | 777 | 1051.3 | 74 | | 69-79 | 696 | 909.11 | 77 | | 71-82 |
| Genitourinary diseases | 580-629 | 221 | 316.94 | 70 | | 61-80 | 186 | 262.39 | 71 | | 61-82 |
| All accidents and violence | 800-999.8 | 1459 | 1825.77 | 80 | | 76-84 | 912 | 1106.62 | 82 | | 77-88 |
| Unknown causes | 799.9, 999.9 | 527 |  |  | |  | 431 |  |  | |  |

*Notes*

1. Excluding the first 10 years after the start of radiation work (2 years for leukaemia).
2. Number expected based on England and Wales rates (not adjusted for social class)
3. Confidence interval
4. Excluding secondary liver cancer
5. Based on a 10 year lag

| **TABLE S2 Test for trend in mortality with dose by cause of death (lagged by 10 years, except for leukaemia where a 2-year lag is used)** | | | | | | | | | | | | | | |  | |
| --- | --- | --- | --- | --- | --- | --- | --- | --- | --- | --- | --- | --- | --- | --- | --- | --- |
|  |  |  |  |  | | Malignant neoplasms | | |  |  |  |  | |  | | |
| Dose (mSv) | *Number of deaths* | All Causes | All known causes excluding malignant neoplasms | All neoplasms | | All malignant neoplasms | | All malignant neoplasms excluding leukaemia | All malignant neoplasms excluding lung, pleura and leukaemia | Mouth, tongue and pharynx | Oesophagus | Stomach | | Large intestine | | |
| <10 | *Obs* | 11836 | 7643 | 3982 | | 3917 | | 3803 | 2626 | 60 | 186 | 229 | | 310 | | |
|  | *Exp* | 11877.37 | 7653.89 | 4036.13 | 3969.54 | | | 3854.81 | 2655.18 | 53.09 | 176.98 | 243.38 | | 300.33 | | |
| 10- | *Obs* | 2920 | 1908 | 981 | 967 | | | 940 | 662 | 16 | 40 | 70 | | 79 | | |
|  | *Exp* | 2937.61 | 1935.59 | 972.91 | 955.77 | | | 926.13 | 635.46 | 11.81 | 39.89 | 67.43 | | 72.89 | | |
| 20- | *Obs* | 3693 | 2454 | 1211 | 1182 | | | 1142 | 777 | 5 | 53 | 89 | | 79 | | |
|  | *Exp* | 3726.4 | 2459.39 | 1228.2 | 1208.08 | | | 1169.99 | 800.64 | 14.74 | 52.1 | 87.37 | | 92.02 | | |
| 50- | *Obs* | 2082 | 1360 | 711 | | | 703 | 682 | 450 | 7 | 20 | 62 | 55 | | |  |
|  | *Exp* | 2067.73 | 1362.25 | 683.15 | | | 672.26 | 650.98 | 446.16 | 8.08 | 30.15 | 50.2 | 52.56 | | |  |
| 100- | *Obs* | 1380 | 912 | 461 | | | 454 | 442 | 294 | 4 | 13 | 30 | 31 | | |  |
|  | *Exp* | 1365.53 | 903.08 | 448.53 | | | 441.33 | 428.29 | 293.74 | 5.33 | 20.82 | 34.08 | 36.19 | | |  |
| 200- | *Obs* | 914 | 614 | 291 | | | 288 | 277 | 187 | 4 | 21 | 24 | 20 | | |  |
|  | *Exp* | 855.01 | 568.77 | 278.85 | | | 274.78 | 267.02 | 179.99 | 3.3 | 12.86 | 22.02 | 21.3 | | |  |
| 400+ | *Obs* | 501 | 320 | 175 | | | 173 | 169 | 122 | 2 | 8 | 14 | 14 | | |  |
|  | *Exp* | 496.34 | 328.03 | 164.23 | | | 162.23 | 157.79 | 106.84 | 1.64 | 8.19 | 13.52 | 12.71 | | |  |
| Total deaths in informative strata |  | 2323326 | 15211 | 7812 | | | 7684 | 7455 | 5118 | 98 | 341 | 518 | 588 | | |  |
| Score statistic |  | 1.66 | 0.86 | 1.74 | | | 1.8 | 1.75 | 1.75 | -0.16 | 0.23 | 0.58 | -0.27 | | |  |
| 1-sided p-value |  | 0.049 | 0.194 | 0.041 | | | 0.036 | 0.04 | 0.04 | 0.536 | 0.407 | 0.282 | 0.606 | | |  |
| 2-sided p-value |  | 0.098 | 0.388 | 0.081 | | | 0.073 | 0.08 | 0.081 | 0.928 | 0.815 | 0.564 | 0.788 | | |  |
| ERR Sv^-1^ |  | 0.145 | 0.093 | 0.268 | | | 0.279 | 0.275 | 0.323 | -0.162 | 0.146 | 0.336 | -0.126 | | |  |
| 90% CI |  | (0.00, 0.3) | (-0.08, 0.28) | (0.01, 0.55) | | | (0.02, 0.56) | (0.02, 0.56) | (0.02, 0.67) | (-1.26, 2.22 ) | (-0.72, 1.42) | (-0.51, 1.58) | (-0.75, 0.77) | | |  |
| 95% CI |  | (-0.03, 0.33) | (-0.11, 0.32) | (-0.03, 0.61) | | | (-0.02, 0.62) | (-0.03, 0.62) | (-0.04, 0.74) | (-1.38, 2.86 ) | (-0.84, 1.72) | (-0.63, 1.88) | (-0.84, 0.98) | | |  |

| **TABLE S2 Test for trend in mortality with dose by cause of death (lagged by 10 years, except for leukaemia where a 2-year lag is used) (cont.)** | | | | | | | | | | | |  |
| --- | --- | --- | --- | --- | --- | --- | --- | --- | --- | --- | --- | --- |
|  |  | Malignant neoplasms *continued* | | |  |  |  |  |  |  |  | |
| Dose (mSv) | *Number of deaths* | Rectum | Liver[[1]](#footnote-2) | Primary Liver | Gallbladder | Pancreas | Larynx | Trachea, bronchus and lung | Pleura | Bone | Connective and soft tissue | |
| <10 | *Obs* | 130 | 46 | 25 | 14 | 171 | 33 | 1125 | 52 | 5 | 21 | |
|  | *Exp* | 150.51 | 48.61 | 22.42 | 15.11 | 175.88 | 35.84 | 1146.93 | 52.69 | 4.51 | 19.18 | |
| 10- | *Obs* | 41 | 10 | 3 | 5 | 43 | 9 | 270 | 8 | 1 | 3 | |
|  | *Exp* | 36.87 | 11.69 | 5.23 | 3.81 | 40.46 | 8.41 | 278.71 | 11.96 | 1.07 | 3.31 | |
| 20- | *Obs* | 59 | 16 | 5 | 8 | 47 | 12 | 349 | 16 | 1 | 2 | |
|  | *Exp* | 47.59 | 13.33 | 5.68 | 4.93 | 50.27 | 9.97 | 353.4 | 15.95 | 1.06 | 3.63 | |
| 50- | *Obs* | 28 | 5 | 3 | 0 | 34 | 1 | 224 | 8 | 1 | 3 | |
|  | *Exp* | 28.48 | 7.49 | 3.22 | 2.52 | 27.56 | 5.15 | 194.86 | 9.96 | 0.53 | 2.35 | |
| 100- | *Obs* | 20 | 8 | 3 | 1 | 19 | 5 | 136 | 12 | 0 | 2 | |
|  | *Exp* | 19.42 | 4.61 | 1.99 | 1.47 | 18.03 | 3.63 | 127.12 | 7.43 | 0.35 | 1.47 | |
| 200- | *Obs* | 14 | 3 | 1 | 1 | 10 | 3 | 82 | 8 | 0 | 0 | |
|  | *Exp* | 13.07 | 2.13 | 0.98 | 0.74 | 11.39 | 2.4 | 81.71 | 5.32 | 0.3 | 0.67 | |
| 400+ | *Obs* | 11 | 1 | 0 | 0 | 6 | 4 | 44 | 3 | 0 | 0 | |
|  | *Exp* | 7.05 | 1.13 | 0.47 | 0.42 | 6.42 | 1.59 | 47.28 | 3.68 | 0.19 | 0.39 | |
| Total deaths in informative strata |  | 303 | 89 | 40 | 29 | 330 | 67 | 2230 | 107 | 8 | 31 | |
| Score statistic |  | 1.93 | 0.800 | -0.4 | -0.76 | -0.06 | 2.13 | 0.35 | 0.81 | -0.93 | -0.87 | |
| 1-sided p-value |  | 0.027 | 0.208 | 0.619 | 0.75 | 0.525 | 0.026 | 0.363 | 0.209 | 0.811 | 0.787 | |
| 2-sided p-value |  | 0.054 | 0.415 | 0.761 | 0.499 | 0.95 | 0.052 | 0.726 | 0.417 | 0.377 | 0.426 | |
| ERR Sv^-1^ |  | 1.687 | 0.800 | -1.500 | <-1.929 | -0.049 | 4.071 | 0.106 | 1.311 | <-1.929 | <-1.929 | |
| 90% CI |  | (0.19, 4.12) | (-1.19, 8.28 ) | (<-1.93, 6.3) | (<-1.93, 4.69) | (-1.00, 1.64) | (0.57, 12.02) | (-0.35, 0.67) | (-0.87, 5.69) | (<-1.93, 12.1) | (<-1.93, 4.76) | |
| 95% CI |  | (-0.02, 4.73) | (-1.43, 10 ) | (<-1.93, 8.56) | (<-1.93, 7.6) | (-1.11, 2.07) | (0.18, 14.46) | (-0.43, 0.79) | (-1.09, 6.87) | (<-1.93, 28.51) | (<-1.93, 7.49) | |

| **TABLE S2 Tests for trend in mortality with dose by cause of death (lagged by 10 years, except for leukaemia where a 2-year lag is used) (cont.)** | | | | | | | | | | | | | | | | | |  | |
| --- | --- | --- | --- | --- | --- | --- | --- | --- | --- | --- | --- | --- | --- | --- | --- | --- | --- | --- | --- |
|  |  | Malignant neoplasms *continued* | | |  | |  |  | |  | |  | | |  |  | | | |
| Dose (mSv) | *Number of deaths* | All skin | Female breast | Uterus | | Ovary | Prostate | | Testis | | Bladder | | Kidney | All Brain | | | Thyroid | |  |
| <10 | *Obs* | 57 | 41 | 13 | | 12 | 348 | | 7 | | 148 | | 100 | 154 | | | 8 | |  |
|  | *Exp* | 55.12 | 42.08 | 13.36 | | 13.25 | 347.66 | | 7.33 | | 148.22 | | 99.28 | 156.67 | | | 9.46 | |  |
| 10- | *Obs* | 7 | 7 | 3 | | 2 | 90 | | 1 | | 42 | | 32 | 40 | | | 2 | |  |
|  | *Exp* | 9.81 | 7.12 | 2.78 | | 2.16 | 88.84 | | 1.32 | | 38.93 | | 22.65 | 35.33 | | | 1.71 | |  |
| 20- | *Obs* | 12 | 5 | 1 | | 4 | 108 | | 1 | | 45 | | 25 | 46 | | | 4 | |  |
|  | *Exp* | 12.58 | 5.03 | 2.23 | | 2.01 | 116.26 | | 1.71 | | 49.84 | | 28.8 | 39.19 | | | 2.67 | |  |
| 50- | *Obs* | 8 | 3 | 1 | | 0 | 69 | | 2 | | 27 | | 19 | 15 | | | 1 | |  |
|  | *Exp* | 6.7 | 1.14 | 0.45 | | 0.33 | 66.09 | | 1.13 | | 27.14 | | 16.09 | 21.87 | | | 1.38 | |  |
| 100- | *Obs* | 4 | 0 | 0 | | 0 | 42 | | 1 | | 17 | | 5 | 16 | | | 1 | |  |
|  | *Exp* | 4.51 | 0.53 | 0.15 | | 0.16 | 41.5 | | 0.85 | | 17.81 | | 10.44 | 13.56 | | | 0.94 | |  |
| 200- | *Obs* | 3 | 0 | 1 | | 0 | 25 | | 1 | | 14 | | 3 | 6 | | | 0 | |  |
|  | *Exp* | 2.86 | 0.1 | 0.01 | | 0.09 | 25.37 | | 0.49 | | 11.82 | | 6.3 | 7.61 | | | 0.58 | |  |
| 400+ | *Obs* | 2 | 0 | 0 | | 0 | 20 | | 0 | | 8 | | 3 | 1 | | | 1 | |  |
|  | *Exp* | 1.43 | 0 | 0.01 | | 0 | 16.28 | | 0.18 | | 7.24 | | 3.44 | 3.75 | | | 0.26 | |  |
| Total deaths in informative strata |  | 93 | 56 | 19 | | 18 | 702 | | 13 | | 301 | | 187 | 278 | | | 17 | |  |
| Score statistic |  | 0.44 | 0.18 | 2.76 | | -0.11 | 0.88 | | 0.38 | | 0.54 | | -1.56 | -1.54 | | | 0.91 | |  |
| 1-sided p-value |  | 0.316 | 0.37 | 0.016 | | 0.418 | 0.191 | | 0.301 | | 0.296 | | 0.941 | 0.938 | | | 0.177 | |  |
| 2-sided p-value |  | 0.632 | 0.736 | 0.03 | | 0.818 | 0.381 | | 0.602 | | 0.591 | | 0.118 | 0.125 | | | 0.352 | |  |
| ERR Sv^-1^ |  | 0.637 | 2.285 | 17.805 | | <-1.929 | 0.416 | | 3.291 | | 0.400 | | -1.028 | -1.362 | | | 3.124 | |  |
| 90% CI |  | (-1.07, 4.78) | (<-1.93, 30.37) | (<-1.93, 72.27) | | (<-1.93, 89.13) | (-0.31, 1.41) | | (<-1.93, 42.71) | | (-0.64, 2.07) | | (-1.52, 0.08) | (-1.82, 0.13) | | | (-0.88, 44.89) | |  |
| 95% CI |  | (-1.23, 5.98) | (<-1.93, 38.21) | (<-1.93, 91.96) | | (<-1.93, 121.76) | (-0.42, 1.64) | | (<-1.93, 59.25) | | (-0.78, 2.48) | | (-1.57, 0.39) | (-1.85, 0.55) | | | (-1.09, 68.13) | |  |

| **TABLE S2 Tests for trend in mortality with dose by cause of death (lagged by 10 years, except for leukaemia where a 2-year lag is used) (cont.)** | | | | | | | | | | | | |
| --- | --- | --- | --- | --- | --- | --- | --- | --- | --- | --- | --- | --- |
|  |  | Malignant neoplasms *continued* | | |  |  |  |  |  |  | Non-Malignant Diseases | |
| Dose (mSv) | *Number of deaths* | Ill-defined and secondary cancers | Lymphatic or haematopoietic | Non-Hodgkin lymphoma | Hodgkin lymphoma | Multiple myeloma | All leukaemia | Leukaemia excluding chronic lymphatic | Malignant neoplasms strongly related to smoking |  | Coronary heart disease | Bronchitis, emphysema & chronic obstructive disease |
| <10 | *Obs* | 326 | 308 | 118 | 18 | 58 | 143 | 115 | 1875 |  | 3526 | 495 |
|  | *Exp* | 320.58 | 318.26 | 122.36 | 19.67 | 61.5 | 135.12 | 106.45 | 1888.9 |  | 3540.69 | 472.02 |
| 10- | *Obs* | 60 | 83 | 40 | 3 | 13 | 26 | 18 | 460 |  | 920 | 130 |
|  | *Exp* | 75.76 | 79.19 | 31.07 | 4.19 | 14.28 | 33.98 | 24.11 | 452.84 |  | 908.49 | 128.78 |
| 20- | *Obs* | 94 | 103 | 36 | 7 | 20 | 43 | 25 | 552 |  | 1112 | 172 |
|  | *Exp* | 99.46 | 97.71 | 37.28 | 4.7 | 17.64 | 43.32 | 29.87 | 575.06 |  | 1169.91 | 163.66 |
| 50- | *Obs* | 56 | 46 | 11 | 2 | 12 | 21 | 13 | 340 |  | 664 | 77 |
|  | *Exp* | 54.22 | 52.8 | 20.33 | 2.02 | 9.16 | 24.86 | 17.24 | 318.99 |  | 662.64 | 88.35 |
| 100- | *Obs* | 49 | 36 | 17 | 3 | 4 | 15 | 11 | 211 |  | 467 | 54 |
|  | *Exp* | 34.75 | 33.95 | 13.72 | 1.29 | 5.89 | 15.48 | 10.59 | 210.62 |  | 446.07 | 58.17 |
| 200- | *Obs* | 18 | 24 | 9 | 0 | 4 | 13 | 10 | 145 |  | 322 | 30 |
|  | *Exp* | 20.88 | 19.52 | 7.86 | 0.79 | 3.12 | 8.76 | 6.04 | 135.11 |  | 280.54 | 37.12 |
| 400+ | *Obs* | 16 | 12 | 6 | 0 | 2 | 6 | 6 | 78 |  | 157 | 14 |
|  | *Exp* | 13.35 | 10.57 | 4.38 | 0.33 | 1.41 | 5.48 | 3.69 | 79.47 |  | 159.66 | 23.9 |
| Total deaths in informative strata |  | 619 | 612 | 237 | 33 | 113 | 267 | 198 | 3661 |  | 7168 | 972 |
| Score statistic |  | 1.17 | 1.08 | 0.89 | -0.31 | 0.75 | 0.76 | 1.73 | 0.58 |  | 1.62 | -3.19 |
| 1-sided p-value |  | 0.121 | 0.14 | 0.186 | 0.58 | 0.221 | 0.225 | 0.042 | 0.282 |  | 0.053 | 0.999 |
| 2-sided p-value |  | 0.242 | 0.28 | 0.372 | 0.839 | 0.442 | 0.45 | 0.084 | 0.565 |  | 0.105 | 0.001 |
| ERR Sv^-1^ |  | 0.689 | 0.655 | 0.777 | <-1.929 | 1.195 | 0.63 | 1.712 | 0.128 |  | 0.259 | -1.041 |
| 90% CI |  | (-0.23, 1.99) | (-0.28, 1.97) | (-0.50, 2.88) | (<-1.93, 24.24) | (-0.88, 5.96) | (-0.57,2.53) | (0.06, 4.29) | (-0.22, 0.53) |  | (0.00, 0.55) | (-1.35, -0.59) |
| 95% CI |  | (-0.37, 2.29) | (-0.43, 2.28) | (-0.66, 3.4) | (<-1.93, 32.73) | (-1.08, 7.31) | (-0.74,2.98) | (-0.17, 4.92) | (-0.28, 0.62) |  | (-0.05, 0.61) | (-1.40, -0.48) |

| **TABLE S2 Tests for trend in mortality with dose by cause of death (lagged by 10 years, except for leukaemia where a 2-year lag is used) (cont.)** | | | | | | | | | | | |  |
| --- | --- | --- | --- | --- | --- | --- | --- | --- | --- | --- | --- | --- |
|  |  | Non-Malignant Diseases *continued* | | |  |  |  |  |  |  |  | |
| Dose (mSv) | *Number of deaths* | Aortic aneurysm | Non-malignant diseases strongly related to smoking | Circulatory diseases not strongly related to smoking | All circulatory diseases | Cerebrovascular disease | Respiratory diseases not strongly related to smoking | Digestive | Genito-urinary | All accidents and violence | Unknown causes | |
| <10 | *Obs* | 219 | 4240 | 1395 | 5140 | 846 | 446 | 353 | 78 | 541 | 276 | |
|  | *Exp* | 237.35 | 4250.06 | 1399.19 | 5177.23 | 861.44 | 441.08 | 361.14 | 78.93 | 545.57 | 253.94 | |
| 10- | *Obs* | 50 | 1100 | 359 | 1329 | 234 | 105 | 86 | 28 | 107 | 45 | |
|  | *Exp* | 58.3 | 1095.57 | 380.38 | 1347.17 | 241.29 | 113.15 | 76.79 | 22.92 | 102.3 | 46.24 | |
| 20- | *Obs* | 93 | 1377 | 493 | 1698 | 318 | 138 | 106 | 32 | 121 | 57 | |
|  | *Exp* | 73.8 | 1407.37 | 483.91 | 1727.62 | 312.39 | 146.01 | 95.22 | 30.97 | 116.07 | 58.93 | |
| 50- | *Obs* | 47 | 788 | 258 | 969 | 169 | 80 | 43 | 14 | 69 | 19 | |
|  | *Exp* | 41.52 | 792.51 | 261.54 | 965.7 | 169.19 | 78.55 | 52.06 | 16.93 | 60.63 | 33.22 | |
| 100- | *Obs* | 28 | 549 | 181 | 676 | 127 | 50 | 28 | 9 | 28 | 14 | |
|  | *Exp* | 27.64 | 531.88 | 173.18 | 646.89 | 113.63 | 50.44 | 33.9 | 11.11 | 37.59 | 21.12 | |
| 200- | *Obs* | 24 | 376 | 122 | 468 | 84 | 35 | 19 | 6 | 19 | 12 | |
|  | *Exp* | 16.17 | 333.82 | 112.55 | 409.26 | 75.42 | 31.07 | 19.92 | 7.31 | 20.94 | 11.47 | |
| 400+ | *Obs* | 5 | 176 | 67 | 229 | 39 | 24 | 15 | 6 | 7 | 8 | |
|  | *Exp* | 11.23 | 194.79 | 64.25 | 235.13 | 43.64 | 17.7 | 10.96 | 4.84 | 8.9 | 6.08 | |
| Total deaths in informative strata |  | 466 | 8606 | 2875 | 10509 | 1817 | 878 | 650 | 173 | 892 | 431 | |
| Score statistic |  | -0.132 | 0.35 | 1.12 | 1.88 | 0.49 | 1.76 | 0.49 | -0.11 | -1.13 | -0.6 | |
| 1-sided p-value |  | 0.563 | 0.364 | 0.132 | 0.03 | 0.31 | 0.04 | 0.314 | 0.544 | 0.871 | 0.727 | |
| 2-sided p-value |  | 0.874 | 0.729 | 0.265 | 0.059 | 0.621 | 0.079 | 0.627 | 0.912 | 0.258 | 0.546 | |
| ERR Sv^-1^ |  | -0.132 | 0.050 | 0.280 | 0.251 | 0.161 | 0.799 | 0.237 | -0.082 | -0.593 | -0.305 | |
| 90% CI |  | (-1.17, 1.53) | (-0.18, 0.3) | (-0.12, 0.75) | (0.03, 0.49) | (-0.34, 0.77) | (0.04, 1.79) | (-0.47, 1.25) | (-0.96, 1.54) | (-1.18, 0.33) | (-0.93, 0.64) | |
| 95% CI |  | (-1.29, 1.92) | (-0.22, 0.35) | (-0.19, 0.85) | (-0.01, 0.54) | (-0.42, 0.91) | (-0.08, 2.01) | (-0.58, 1.48) | (-1.07, 1.97) | (-1.26, 0.55) | (-1.02, 0.87) | |

| **TABLE S3 Excess relative risk (ERR) per Sv estimates for the main leukaemia subtypes** | | | | | | | | | | | |
| --- | --- | --- | --- | --- | --- | --- | --- | --- | --- | --- | --- |
|  | Mortality | | | | |  | Incidence | | | | |
|  |  |  |  | p-value | |  |  |  |  | p-value | |
| Leukaemia subtype | Deaths | ERR Sv^-1^ | (90%CI) | 1-sided | 2-sided |  | Cases | ERR Sv^-1^ | (90%CI) | 1-sided | 2-sided |
| Acute myeloid | 102 | 1.215 | (-1.23, 5.69) | 0.239 | 0.479 |  | 109 | 0.616 | (-1.45, 4.83) | 0.362 | 0.724 |
| Chronic myeloid | 44 | 3.266 | (0.44, 9.28) | 0.027 | 0.054 |  | 59 | 4.079 | (0.88, 11.24) | 0.011 | 0.022 |
| Acute lymphatic | 15 | 7.786 | (<-1.92, 89.52) | 0.303 | 0.606 |  | 19 | 8.801 | (<-1.92, 61.8) | 0.203 | 0.405 |
| Chronic lymphatic | 69 | <-1.919 | (<-1.92, 1.23) | 0.884 | 0.232 |  | 128 | -0.117 | (-1.42, 2.71) | 0.538 | 0.925 |
| All leukaemias excluding chronic lymphatic | 198 | 1.712 | (0.06, 4.29) | 0.042 | 0.084 |  | 234 | 1.782 | (0.17, 4.36) | 0.03 | 0.06 |
| All leukaemias | 267 | 0.63 | (-0.57, 2.53) | 0.225 | 0.45 |  | 362 | 1.011 | (-0.18, 2.79) | 0.09 | 0.18 |

| **TABLE S4 Tests for trend in cancer incidence with dose by diagnosis (lagged by 10 years, except for leukaemia where a 2-year lag is used)** | | | | | | | | | | | |  |
| --- | --- | --- | --- | --- | --- | --- | --- | --- | --- | --- | --- | --- |
| Dose (mSv) | *Number of cases* | All neoplasms | All malignant neoplasms | All malignant neoplasms excluding leukaemia | All malignant neoplasms excluding lung, pleura and leukaemia | Mouth, tongue and pharynx | Oesophagus | Stomach | Large intestine | Rectum | Liver[[2]](#footnote-3) | |
| <10 | *Obs* | 6411 | 5918 | 5756 | 4521 | 104 | 159 | 305 | 472 | 295 | 46 | |
|  | *Exp* | 6504.56 | 5993.79 | 5832.14 | 4580.49 | 100.2 | 158.08 | 306.97 | 469.91 | 312.72 | 47.44 | |
| 10- | *Obs* | 1463 | 1377 | 1346 | 1066 | 23 | 36 | 82 | 120 | 69 | 7 | |
|  | *Exp* | 1443.48 | 1357.39 | 1318.99 | 1019.79 | 19.55 | 36.11 | 78.79 | 109.56 | 68.35 | 10.89 | |
| 20- | *Obs* | 1786 | 1665 | 1612 | 1238 | 13 | 49 | 95 | 122 | 95 | 16 | |
|  | *Exp* | 1806.81 | 1703.95 | 1654.42 | 1273.93 | 23.18 | 45.49 | 102.77 | 136.88 | 89.52 | 12.98 | |
| 50- | *Obs* | 1029 | 976 | 950 | 709 | 18 | 22 | 66 | 90 | 54 | 8 | |
|  | *Exp* | 990.96 | 932.93 | 906.18 | 697.01 | 12.57 | 25.49 | 57.01 | 78.96 | 50.94 | 7.49 | |
| 100- | *Obs* | 682 | 640 | 623 | 474 | 7 | 14 | 33 | 50 | 33 | 7 | |
|  | *Exp* | 643.8 | 605.18 | 587.83 | 450.97 | 8.19 | 17.56 | 37.12 | 53.6 | 32.53 | 4.17 | |
| 200- | *Obs* | 382 | 364 | 349 | 259 | 6 | 13 | 21 | 22 | 24 | 2 | |
|  | *Exp* | 389.07 | 367.05 | 356.33 | 269.83 | 4.96 | 10.95 | 22.7 | 31.97 | 20.6 | 2.08 | |
| 400+ | *Obs* | 243 | 225 | 219 | 176 | 0 | 7 | 16 | 23 | 16 | 0 | |
|  | *Exp* | 217.32 | 204.71 | 199.11 | 150.98 | 2.36 | 6.31 | 12.65 | 18.11 | 11.34 | 0.94 | |
| Total cases in informative strata |  | 11996 | 11165 | 10855 | 8443 | 171 | 300 | 618 | 899 | 586 | 86 | |
| Score statistic |  | 2.33 | 2.09 | 1.96 | 2.01 | -1.11 | 0.22 | 0.6 | -0.07 | 2.05 | -0.09 | |
| 1-sided p-value |  | 0.01 | 0.018 | 0.025 | 0.022 | 0.866 | 0.414 | 0.274 | 0.527 | 0.02 | 0.513 | |
| 2-sided p-value |  | 0.02 | 0.036 | 0.05 | 0.045 | 0.268 | 0.828 | 0.549 | 0.947 | 0.041 | 0.973 | |
| ERR Sv^-1^ |  | 0.302 | 0.281 | 0.266 | 0.305 | -1.756 | 0.154 | 0.305 | -0.026 | 1.307 | -0.09 | |
| 90% CI |  | (0.08, 0.54) | (0.06, 0.53) | (0.04, 0.51) | (0.05, 0.58) | (<-1.93, 0.98 ) | (-0.79, 1.68) | (-0.44, 1.37) | (-0.56, 0.71) | (0.21, 2.85) | (<-1.93, 6.58 ) | |
| 95% CI |  | (0.05, 0.59) | (0.02, 0.57) | (0.00, 0.56) | (0.01, 0.64) | (<-1.93, 1.63 ) | (-0.91, 2.06) | (-0.55, 1.62) | (-0.65, 0.88) | (0.04, 3.2) | (<-1.93, 8.39 ) | |

| **TABLE S4 Tests for trend in cancer incidence with dose by diagnosis (lagged by 10 years, except for leukaemia where a 2-year lag is used) (cont.)** | | | | | | | | | | | |
| --- | --- | --- | --- | --- | --- | --- | --- | --- | --- | --- | --- |
| Dose (mSv) | *Number of cases* | Primary Liver | Gallbladder | Pancreas | Larynx | Trachea, bronchus and lung | Pleura | Bone | Connective and soft tissue | All skin | Malignant melanoma |
| <10 | *Obs* | 35 | 22 | 164 | 93 | 1148 | 87 | 7 | 38 | 313 | 160 |
|  | *Exp* | 30.81 | 23.42 | 172.34 | 85.53 | 1149.49 | 102.15 | 8.72 | 34.73 | 328.03 | 166.09 |
| 10- | *Obs* | 2 | 8 | 44 | 16 | 255 | 25 | 3 | 5 | 71 | 26 |
|  | *Exp* | 7.11 | 5.67 | 39.93 | 19.61 | 278.17 | 21.04 | 2.38 | 6.29 | 68.78 | 28.93 |
| 20- | *Obs* | 8 | 11 | 44 | 22 | 343 | 31 | 3 | 6 | 91 | 37 |
|  | *Exp* | 8.45 | 7.12 | 48.84 | 24.92 | 352.4 | 28.08 | 2.61 | 7.51 | 85.77 | 31.97 |
| 50- | *Obs* | 5 | 0 | 37 | 9 | 223 | 18 | 2 | 6 | 47 | 18 |
|  | *Exp* | 4.91 | 3.99 | 26.32 | 14.09 | 193.01 | 16.16 | 1.55 | 4.01 | 47.18 | 16.33 |
| 100- | *Obs* | 4 | 1 | 18 | 12 | 131 | 18 | 1 | 3 | 29 | 11 |
|  | *Exp* | 2.6 | 2.57 | 16.58 | 10.29 | 125.42 | 11.44 | 0.89 | 2.87 | 29.65 | 10.01 |
| 200- | *Obs* | 2 | 2 | 7 | 7 | 83 | 7 | 1 | 0 | 19 | 5 |
|  | *Exp* | 1.39 | 1.41 | 10.38 | 6.89 | 79.76 | 6.73 | 0.6 | 1.75 | 17.45 | 5.11 |
| 400+ | *Obs* | 0 | 1 | 6 | 6 | 39 | 4 | 0 | 0 | 17 | 4 |
|  | *Exp* | 0.73 | 0.81 | 5.62 | 3.67 | 43.74 | 4.39 | 0.25 | 0.83 | 10.14 | 2.56 |
| Total cases in informative strata |  | 56 | 45 | 320 | 165 | 2222 | 190 | 17 | 58 | 587 | 261 |
| Score statistic |  | -0.2 | -0.15 | 0.09 | 0.95 | 0.16 | 0.91 | 0.14 | -1.55 | 2.34 | 1.13 |
| 1-sided p-value |  | 0.548 | 0.513 | 0.463 | 0.172 | 0.434 | 0.182 | 0.4 | 0.955 | 0.01 | 0.128 |
| 2-sided p-value |  | 0.905 | 0.973 | 0.926 | 0.343 | 0.869 | 0.363 | 0.799 | 0.09 | 0.019 | 0.257 |
| ERR Sv^-1^ |  | -0.651 | -0.226 | 0.078 | 0.839 | 0.051 | 1.354 | 1.177 | <-1.934 | 1.466 | 1.390 |
| 90% CI |  | (<-1.93, 5.96) | (-1.50, 3.88) | (-0.95, 2) | (-0.46, 3.05) | (-0.41, 0.62) | (-0.71, 5.51) | (<-1.93, 36.34) | (<-1.93, 0.3) | (0.36, 3.03) | (-0.43, 4.74) |
| 95% CI |  | (<-1.93, 7.73) | (-1.59, 5.29) | (-1.07, 2.51) | (-0.63, 3.61) | (-0.49, 0.74) | (-0.94, 6.61) | (<-1.93, 52.16) | (<-1.93, 1.42) | (0.19, 3.39) | (-0.65, 5.6) |

| **TABLE S4 Tests for trend in cancer incidence with dose by diagnosis (lagged by 10 years, except for leukaemia where a 2-year lag is used) (cont.)** | | | | | | | | | | | |  |
| --- | --- | --- | --- | --- | --- | --- | --- | --- | --- | --- | --- | --- |
| Dose (mSv) | *Number of cases* | Non-melanoma skin cancer | Female breast | Uterus | Ovary | Prostate | Testis | Bladder | Kidney | All Brain | Thyroid | |
| <10 | *Obs* | 153 | 110 | 46 | 10 | 758 | 84 | 399 | 163 | 199 | 34 | |
|  | *Exp* | 161.94 | 114.38 | 47.02 | 10.48 | 774.09 | 79.37 | 392.51 | 164.58 | 197.85 | 35.4 | |
| 10- | *Obs* | 45 | 16 | 5 | 2 | 213 | 7 | 89 | 48 | 48 | 7 | |
|  | *Exp* | 39.85 | 17.51 | 5.81 | 1.94 | 187.54 | 11.95 | 92.03 | 35.11 | 40.38 | 5.71 | |
| 20- | *Obs* | 54 | 20 | 4 | 3 | 239 | 10 | 106 | 32 | 45 | 6 | |
|  | *Exp* | 53.8 | 13.46 | 3.7 | 1.9 | 248.92 | 12.64 | 116.53 | 44.1 | 45.15 | 6.29 | |
| 50- | *Obs* | 29 | 3 | 2 | 0 | 135 | 8 | 63 | 29 | 21 | 2 | |
|  | *Exp* | 30.85 | 3.23 | 0.9 | 0.43 | 137.45 | 6.41 | 64.63 | 23.79 | 25.11 | 3.25 | |
| 100- | *Obs* | 18 | 2 | 0 | 0 | 97 | 3 | 43 | 12 | 14 | 3 | |
|  | *Exp* | 19.64 | 1.8 | 0.46 | 0.2 | 87.09 | 3.26 | 42.14 | 15.13 | 15.49 | 1.88 | |
| 200- | *Obs* | 14 | 0 | 1 | 0 | 47 | 4 | 30 | 8 | 7 | 0 | |
|  | *Exp* | 12.34 | 0.49 | 0.11 | 0.05 | 50.15 | 1.71 | 25.76 | 9.09 | 8.7 | 1 | |
| 400+ | *Obs* | 13 | 0 | 0 | 0 | 27 | 0 | 18 | 4 | 3 | 2 | |
|  | *Exp* | 7.58 | 0.12 | 0 | 0 | 30.77 | 0.66 | 14.4 | 4.21 | 4.32 | 0.47 | |
| Total cases in informative strata |  | 326 | 151 | 58 | 15 | 1516 | 116 | 748 | 296 | 337 | 54 | |
| Score statistic |  | 2.05 | -0.03 | 1.74 | -0.39 | -0.53 | 0.37 | 1.28 | -0.55 | -1.25 | 1.48 | |
| 1-sided p-value |  | 0.02 | 0.511 | 0.057 | 0.587 | 0.701 | 0.33 | 0.1 | 0.708 | 0.895 | 0.079 | |
| 2-sided p-value |  | 0.04 | 0.978 | 0.112 | 0.826 | 0.599 | 0.66 | 0.199 | 0.584 | 0.21 | 0.157 | |
| ERR Sv^-1^ |  | 1.497 | -0.228 | 10.523 | <-1.934 | -0.180 | 1.018 | 0.646 | -0.411 | -0.882 | 3.236 | |
| 90% CI |  | (0.23, 3.4) | (<-1.93, 14.49) | (0.27, 39.4) | (<-1.93, 61.13) | (-0.65, 0.43) | (<-1.93, 7.21) | (-0.15, 1.72) | (-1.22, 1.09) | (-1.49, 0.36) | (-0.19, 13.9) | |
| 95% CI |  | (0.05, 3.85) | (<-1.93, 18.09) | (-0.50, 48.02) | (<-1.93, 88.75) | (-0.73, 0.57) | (<-1.93, 8.85) | (-0.28, 1.96) | (-1.32, 1.48) | (-1.56, 0.69) | (-0.48, 17.51) | |

| **TABLE S4 Tests for trend in cancer incidence with dose by diagnosis (lagged by 10 years, except for leukaemia where a 2-year lag is used) (cont.)** | | | | | | | | | | | |  |
| --- | --- | --- | --- | --- | --- | --- | --- | --- | --- | --- | --- | --- |
| Dose (mSv) | *Number of cases* | Ill-defined and secondary cancers | Lymphatic or haematopoietic | Non-Hodgkin lymphoma | Hodgkin lymphoma | Multiple myeloma | All leukaemia | Leukaemia excluding chronic lymphatic | All malignant neoplasms excluding non-melanoma skin cancer | Malignant neoplasms strongly related to smoking |  | |
| <10 | *Obs* | 299 | 442 | 166 | 43 | 71 | 198 | 135 | 5758 | 2317 |  | |
|  | *Exp* | 287.91 | 456.33 | 169.03 | 42.88 | 82.76 | 190.46 | 130.74 | 5828.11 | 2324.88 |  | |
| 10- | *Obs* | 57 | 97 | 42 | 3 | 21 | 32 | 20 | 1351 | 536 |  | |
|  | *Exp* | 69.83 | 102.02 | 38.46 | 7.16 | 17.99 | 44.48 | 27.73 | 1328.66 | 541.54 |  | |
| 20- | *Obs* | 88 | 138 | 46 | 12 | 27 | 55 | 35 | 1628 | 640 |  | |
|  | *Exp* | 90.36 | 126.62 | 45.86 | 7.89 | 23.35 | 56.19 | 34.49 | 1672.3 | 683.56 |  | |
| 50- | *Obs* | 44 | 56 | 14 | 2 | 14 | 31 | 16 | 959 | 419 |  | |
|  | *Exp* | 50.92 | 66.21 | 23.51 | 4.01 | 11.94 | 31.43 | 18.74 | 916.67 | 376.06 |  | |
| 100- | *Obs* | 51 | 49 | 19 | 6 | 7 | 21 | 12 | 629 | 255 |  | |
|  | *Exp* | 35.03 | 42.64 | 15.27 | 2.63 | 7.39 | 20.37 | 11.67 | 595.18 | 246.74 |  | |
| 200- | *Obs* | 17 | 32 | 12 | 1 | 4 | 16 | 9 | 359 | 161 |  | |
|  | *Exp* | 22.94 | 24.62 | 8.44 | 1.68 | 3.78 | 12.08 | 6.89 | 361.94 | 154.52 |  | |
| 400+ | *Obs* | 15 | 17 | 6 | 0 | 5 | 9 | 7 | 221 | 84 |  | |
|  | *Exp* | 14.01 | 12.57 | 4.43 | 0.76 | 1.78 | 6.99 | 3.74 | 202.14 | 84.7 |  | |
| Total cases in informative strata |  | 571 | 831 | 305 | 67 | 149 | 362 | 234 | 10905 | 4412 |  | |
| Score statistic |  | 0.19 | 2.33 | 1.4 | -0.39 | 2.43 | 1.34 | 1.88 | 1.98 | 0.8 |  | |
| 1-sided p-value |  | 0.425 | 0.01 | 0.081 | 0.626 | 0.008 | 0.09 | 0.03 | 0.024 | 0.213 |  | |
| 2-sided p-value |  | 0.849 | 0.02 | 0.162 | 0.748 | 0.015 | 0.18 | 0.06 | 0.048 | 0.426 |  | |
| ERR Sv^-1^ |  | 0.100 | 1.344 | 1.284 | <-1.934 | 3.597 | 1.011 | 1.782 | 0.267 | 0.170 |  | |
| 90% CI |  | (-0.63, 1.17) | (0.34, 2.67) | (-0.18, 3.53) | (<-1.93, 9.08) | (0.77, 8.94) | (-0.18, 2.79) | (0.17, 4.36) | (0.04, 0.51) | (-0.17, 0.56) |  | |
| 95% CI |  | (-0.74, 1.42) | (0.18, 2.97) | (-0.38, 4.06) | (<-1.93, 12.55) | (0.43, 10.37) | (-0.36, 3.21) | (-0.06, 4.99) | (0.00, 0.56) | (-0.23, 0.64) |  | |

1. Excluding secondary liver cancer. [↑](#footnote-ref-2)
2. Excluding secondary liver cancer. [↑](#footnote-ref-3)
